# Clustering Cities over Features Extracted from Multiple Virtual Sensors Measuring Micro-Level Activity Patterns Allows One to Discriminate Large-Scale City Characteristics

**DOI:** 10.3390/s23115165

**Published:** 2023-05-29

**Authors:** Ricardo Muñoz-Cancino, Sebastián A. Ríos, Manuel Graña

**Affiliations:** 1 Computational Intelligence Group, University of Basque Country, 20018 San Sebastián, Spain; 2 Business Intelligence Research Center (CEINE), Department of Industrial Engineering, University of Chile, Beauchef 851, Santiago 8370456, Chile

**Keywords:** activity patterns, clustering models, geotagged data, City Innovation Index

## Abstract

The impact of micro-level people’s activities on urban macro-level indicators is a complex question that has been the subject of much interest among researchers and policymakers. Transportation preferences, consumption habits, communication patterns and other individual-level activities can significantly impact large-scale urban characteristics, such as the potential for innovation generation of the city. Conversely, large-scale urban characteristics can also constrain and determine the activities of their inhabitants. Therefore, understanding the interdependence and mutual reinforcement between micro- and macro-level factors is critical to defining effective public policies. The increasing availability of digital data sources, such as social media and mobile phones, has opened up new opportunities for the quantitative study of this interdependency. This paper aims to detect meaningful city clusters on the basis of a detailed analysis of the spatiotemporal activity patterns for each city. The study is carried out on a worldwide city dataset of spatiotemporal activity patterns obtained from geotagged social media data. Clustering features are obtained from unsupervised topic analyses of activity patterns. Our study compares state-of-the-art clustering models, selecting the model achieving a 2.7% greater Silhouette Score than the next-best model. Three well-separated city clusters are identified. Additionally, the study of the distribution of the City Innovation Index over these three city clusters shows discrimination of low performing from high performing cities relative to innovation. Low performing cities are identified in one well-separated cluster. Therefore, it is possible to correlate micro-scale individual-level activities to large-scale urban characteristics.

## 1. Introduction

The relationship between the activities conducted by city residents and large-scale city characteristics is a complex phenomenon that has received particular attention from researchers and policymakers [1,2]. Individual-level activities such as transportation preferences [3,4], consumption patterns [5,6], communication patterns and social interactions [7] can significantly impact large-scale city characteristics, such as innovation generation capability. Transportation preferences affect traffic congestion and pollution, affecting the ability of cities to innovate and to compete in a globalized economy. Shopping habits can determine which business areas flourish, thereby boosting the capacity for innovation in related industries. On the other hand, large-scale urban characteristics can constrain or facilitate the activity of their inhabitants. For example, a well-connected public transport network reduces the use of private vehicles. Policies implemented by city managers supporting specific industrial sectors can promote the technical specialization of inhabitants [8]. Furthermore, enhanced access to culture, networking and entertainment can determine social relationships [9,10]. The interplay between micro and macro factors is complicated by their interdependence. The increasing availability of digital data sources, such as social media, mobile phones and a wide variety of virtual sensors, has opened up new opportunities for the quantitative analyisis of the relationship between individual urban activities and large-scale urban characteristics. These data sources are like virtual sensors that capture information on individual behavior providing a more comprehensive and dynamic understanding of urban life [11].

This study investigates the relationship between the activity of the city inhabitants observed through their social media digital traces and large-scale characteristics of the city, such as its capacity to generate innovation. In particular, the following research questions are dealt with in this paper.

1. Can meaningful city categories (clusters) be detected based on micro-level city activity patterns derived from individual citizen activity data?
2. Is there any relationship between the clusters obtained and large-scale city characteristics such as the City Innovation Index [12]?

The study analyzes a worldwide city dataset, obtaining city features based on the decomposition of city spatiotemporal activity patterns of social interactions into latent topic patterns. The dataset covers 17 years of city residents’ digital activity gathered from geotagged social media digital traces. The city features are used to cluster cities with similar behaviors. We explore several state-of-the-art clustering algorithms, selecting the optimal one on the basis of the Silhouette clustering quality measure.

The article is organized as follows: Section 2 provides information on previous works and applications. Section 3 describes the data sources used to build the experimental dataset. Section 4 presents the methodology and experimental setup. Section 5 gives the details of the experimental setup. Section 6 presents and discusses the computational experiment results. Section 7 provides a discussion of results in relation with other recent publications. Finally, Section 8 gives our conclusions and some ideas for future research work.

## 2. Background and Related Work

### 2.1. Large-Scale Urban Characteristics, Innovation and Public Policies

Large-scale urban characteristics, such as the capability for the generation of innovations, are important for urban development and growth [1,13]. Cities that provide environments fostering innovation are more likely to attract investment and new businesses, which leads to higher job market growth and economic prosperity. Boosting the ranking of the city on these large-scale urban characteristics demands a strategic approach that involves public policies and regulation. For example, governments can create incentives and tax reductions [14,15] and invest in developing infrastructure encouraging innovation, such as high-speed internet access [16,17], co-working spaces [18,19], public transportation and social and recreational areas [20]. City Innovation Index rankings allow one to compare the innovation level of cities across the world [21]. These rankings usually involve indicators such as research and development, entrepreneurship, infrastructure, cultural assets, technology transfer and market quality as factors supporting innovation. These rankings make visible and highlight cities’ strengths and weaknesses regarding innovation that provide valuable information for policymakers in charge of developing public policies that help improve weak aspects of the city.

### 2.2. Data-Driven Policy-Making Using Digital Traces

Recent literature has shown the potential of multiple data sources to characterize the interaction between humans and their environments. This research uses social media geotagged digital trace data sources to describe individuals’ behavior. In order to understand their ability to identify novel human behavioral patterns, it is important to distinguish how ubiquitous social media platforms are. Mobile phones are the technology with the fastest adoption rate in history, even in developing countries, where ownership spans more than 91% of the population [22,23]. Over 60% of mobile phone users access social media platforms [24]. This percentage is rising in developed countries; for instance, 90% of the US population is actively engaged in social media [25]. Mobile phone and social media data can characterize activities such as transportation habits or the practice of outdoor sports.

Studies have used geo-crowdsourced data from multiple resources, such as Foursquare [26], geotagged tweets [27,28,29], cell phone records [30,31,32,33,34], geotagged Flickr photos [35] and geotagged Chinese social media messages [36], to understand complex human activity patterns. These patterns are important for public policy decisions related to urban phenomena such as public transport [37], traffic flow [38], flood risk management [39] and urban planning [40,41]. Human behavioral patterns have also been used to measure the effectiveness of pandemic policies [42,43].

However, there are methodological challenges in translating massive data sets into valuable insights, such as aggregating raw data into mobility patterns to monitor quarantine policies [43]. Despite these challenges, the potential of using these data sources to support policy-making is tremendous.

## 3. Data Description

This study uses city activity patterns from the social media dataset described in a recent PhD dissertation [44]. Table 1 gives details of the dataset. It contains over 32 million geotagged urban activity records (tweets, images, check-ins) from various social activity platforms collected over 17 years from 127 cities all over the world. Although each source dataset has a different schema, the available fields are used to avoid duplicate urban activities. The collated data used in this paper are available from the open data repository https://doi.org/10.5281/zenodo.7949307 (accessed on 22 May 2023).

City activity patterns were defined as the count of activity events in a period of a week with a time resolution of one hour; hence, each city has 17×54 weekly activity pattern vectors of size 7×24. Topic analysis has been applied [44] to obtain topic representative activity patterns. Via optimal topic analysis, three such topic representative activity patterns were found for each time slice of 3 years. Each city was characterized by the mean and standard deviation of the parameters, distributing the linear decomposition of its activity patterns into the topic representative activity patterns. In this way, each city is characterized for a k×s matrix, where k=3 is the number of city activity patterns per time slice and s=7 corresponds to the number of time slices.

Additionally, this study uses the City Innovation Index [12], an annual quantitative index to rank the most innovative cities worldwide. The quantitative index is based on cities’ cultural assets, human infrastructure and networked markets. Cultural assets refer to how culture is experienced within cities and considers arts districts, civic institutions, museums, music events, galleries, political protests, books, media, availability of information and sports. Human infrastructure includes the infrastructure deployed in the city for mass transit, finance, universities, hospitals, rail, roads, law, commerce, start-ups, healthcare and telecommunications. Finally, networked markets measure a city’s influence and connections in global markets, considering geography, economics, exports and imports, technology, market size, geo-political aspects and diplomacy.

Finally, this study use the definition of city/town provided by Simplemaps [53], which considers a city/town any inhabited place as determined by U.S. government agencies. The location of cities and their respective centers were obtained from the World Cities Database provided by the same company. We include cities with more than 1 million inhabitants or country capitals in the study. In addition, we define a limit of the ten most populated cities in the country for countries where many cities meet the above-mentioned conditions.

## 4. Methodology and Experimental Setup

### Data Preprocessing

**Figure 1:**
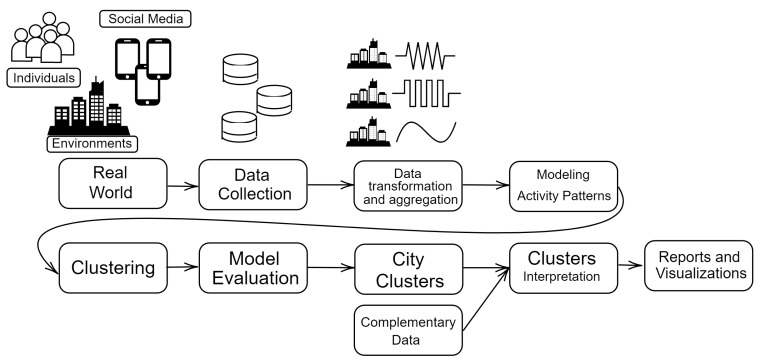
Proposed methodology for discovering city activity patterns and then interpreting the results through city clustering.

**Figure 2:**
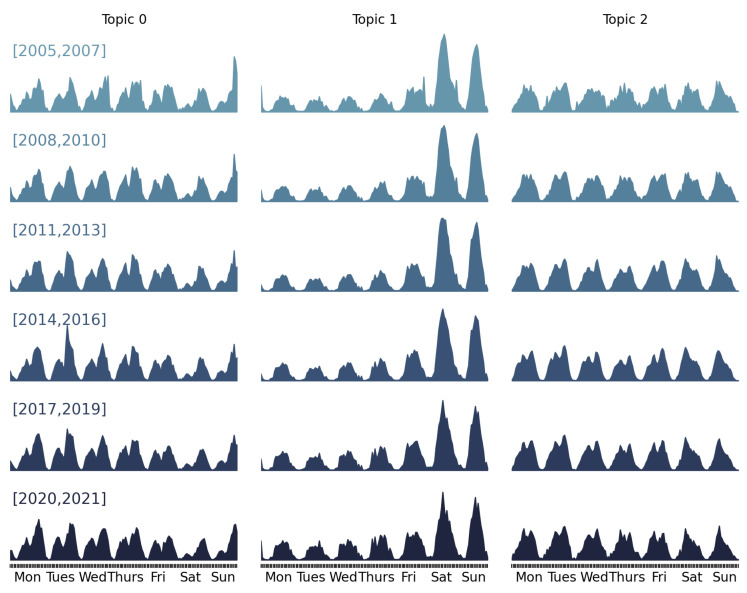
Topic representative activity patterns obtained using Dynamic Topic Models [44] over virtual sensors of digital traces of people activities. Each row corresponds to a time slice of the data; the years are indicated in brackets. Each column corresponds to a topic that has consistent interpretation over time.

The main steps of the computational process leading to the city clustering and interpretation are graphically depicted in Figure 1. The process starts with the integration of real-world data from open data sources described above. A painfully careful and time-consuming effort has been devoted to curating the data, assessing non-duplicate records of events and transforming them into the weekly activity patterns that are the data used in the topic analysis that is used to identify topic representative activity patterns. Here, topics are equated to generic latent activity patterns which can be interpreted as modeling the behavior of some segment of the population. In this computational exploration, we found topic representative activity patterns for business/office work, leisure activities and shopping time. Figure 2 shows the topic representative activity patterns identified in this process in seven time slices of the data, accounting for time variability of the citizens’ behaviors. After the identification of the topic representative activity patterns, each weekly activity pattern can be expressed as a linear combination of them. The coefficients of this linear combination are the features in the latent space describing the weekly activity patterns. The aggregated city features are computed as the means and standard deviations of the weekly activity pattern features. This methodological approach is discussed in detail elsewhere [44]; here, we focus on the last steps of the process, namely the clustering of the cities on the basis of the aggregated city features.

## 5. Experimental Setup

The clustering stage seeks to find groups of cities based on their multi-temporal city activity patterns. State-of-the-art clustering models are evaluated based on multiple model training using bootstrap samples in order to select the best model based on its Silhouette Score [54]. In order to provide an interpretation for the identified city clusters, we study the distribution of the City Innovation Index for each cluster discussing the links between individual-level urban activities and large-scale urban characteristics. The sequence of computational processes is as follows.

- The first step computes city features as the average and standard deviation of the coefficients of the linear decomposition of the weekly activity patterns and sorts them into the topic representative activity patterns extracted from each time slice of the dataset; thus, each city would be described by 2×k×s features, where k=3 is the optimal number of topics selected as described elsewhere [44] and s=7 is the number of time slices. These features are further compressed by averaging over the time slices. Therefore, each city is described by 2×k features for the ensuing clustering experiments.
- The second step is the comparison among state-of-the-art clustering algorithms, namely K-Means, Mini Batch K-Means, Agglomerative Clustering, Spectral Clustering, BIRCH and Gaussian Mixture model. Clustering is repeated 30 time with each algorithm and the number of clusters is set in the range between 2 and 20. The best clustering algorithm and optimal number of clusters are selected using the Silhouette Score over the 30 repetitions. The optimal number of clusters corresponds to the maximum of the average Silhouette Score. The best clustering algorithm is selected based on paired comparisons using the non-parametric Wilcoxon test over the Silhouette Scores achieved along the number of cluster explorations.
- The third step is the computation of the distribution of the City Innovation Index and other large-scale urban indices over the cities included in each cluster of the optimal clustering solution found above. The boxplot visualization of the distribution per cluster shows that the clusters effectively discriminate between cities according to large-scale city indices.

## 6. Results

The presentation of results begins in Section 6.1 with an exploratory analysis of the geotagged digital traces discussing how they represent the human activity in the cities. Then, Section 6.2 reports clustering results based on city features discussed above. This section compares multiple clustering models, selecting the optimal one as discussed above. In Section 6.3, the optimal clusters are characterized based on their geographic location. Finally, Section 6.4 discusses the discrimination of large-scale city indices between the identified optimal clusters.

### 6.1. Data Exploration

**Figure 3:**
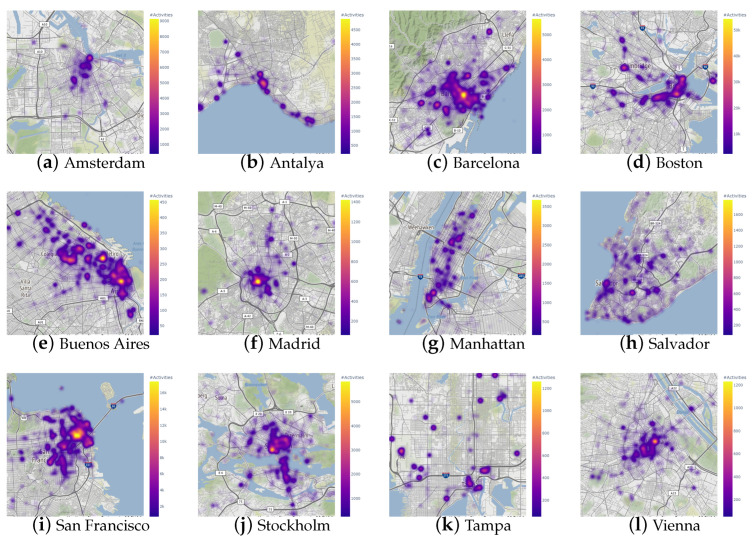
Example of cities included in the dataset and their spatial densities of geotagged digital traces. Yellow indicates a larger activity frequency, while purple indicates a smaller one. Each figure has an independent scale. Map tiles by Stamen Design are under CC BY 3.0, Data by OpenStreetMap contributors are under ODbL.

For visual assessment, Figure 3 shows the density of the geotagged urban activities gathered in the experimental dataset for a sample of cities. For each city shown in the figure, only activities within a 30 km radius are considered to be associated with the city. It can be appreciated that registered activities have a high concentration close to the urban centers of each city. For example, in Amsterdam, we observe an area of high activity in the surroundings of the Amsterdam Centraal Railway Station and other locations of high concentration of people, such as Leidseplein Square, a buzzing nightlife hub surrounded by bars and restaurants. In the case of Manhattan, although the activities are distributed throughout practically the entire island, sectors such as Times Square, the Rockefeller Center and the One World Trade Center stand out as high-activity areas. Less touristic cities, such as Tampa, also show concentrations of activity in areas of importance to the city, as seen in the image: Downtown, The Florida Aquarium and Tampa International Airport.

It is necessary to highlight that the computed weekly activity patterns reflect only the behavior of the population that generated the digital traces. This research does not have demographic information on users to profile them. Therefore, it cannot be ensured that the sample of users is fully representative of the city population.

### 6.2. City Clustering

The study explored the clustering performance of state-of-the-art algorithms, namely K-Means, Mini Batch K-Means, Agglomerative Clustering, Spectral Clustering, BIRCH and Gaussian Mixture model. In order to determine the optimal number of clusters, clustering was carried out varying the number of clusters between 2 and 20 for each algorithm. Furthermore, to assess the robustness of the results, 30 repetitions of the clustering process were carried out for each algorithm and number of clusters.

**Figure 4:**
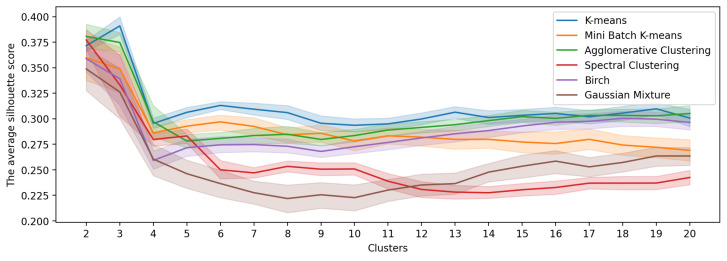
Silhouette Score over groups’ multi-temporal city activity patterns. Each line shows the average Silhouette Score and its 95% confidence interval.

Figure 4 shows the average and 95% confidence interval of the Silhouette Score obtained for each of the models. These results show that the optimal clustering is achieved with K-Means when the optimal number of clusters is set to three. Overall, K-Means showed the highest Silhouette Score values for all settings of the number of clusters. This result was verified by pairwise comparison against the other models using the Wilcoxon test, where K-Means showed statistically significant differences with *p*-values well below 0.05. The details of the comparison using the Wilcoxon test are presented in Table 2. The table shows the result of applying the test to compare the Silhouette Score of the K-Means against the other clustering algorithms. The comparison is presented for models with k=3 and k=2 and the rest are omitted because other scenarios are self-evident.

**Figure 5:**
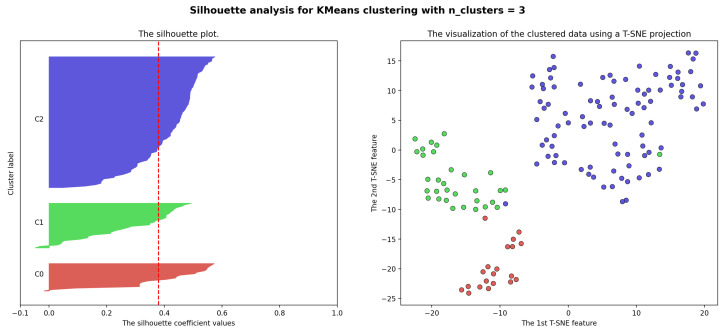
Silhouette analysis for K-Means clustering results with k=3. The **left** figure plots the Silhouette Score for each city in each city in each cluster. The **right** figure is the t-SNE embedding of the clusters.

Additionally, Figure 5 (left) shows the Silhouette Score for each city displayed along the Y axis in decreasing order for each cluster. The Silhouette Score of a city measures its similarity to the cluster it belongs to compared to others. The score ranges from −1 to 1, where 1 indicates that the city is very well fitted to its own cluster and poorly matched to neighboring clusters. A score of −1 indicates the opposite, with the city poorly matched to its own cluster and well-matched to neighboring clusters. A score of 0 indicates that the city is equally similar to its own and neighboring clusters. The optimal K-Means clustering achieves an average score of 0.38, with only a few cities obtaining a score of zero or below. These results indicate that, with a few exceptions, the cities were optimally assigned to their corresponding clusters. On the other hand, Figure 5 (right) shows the spatial embedding of the cities computed via a two-dimensional t-SNE (t-Distributed Stochastic Neighbor Embedding) projection from the six-dimensional space of the city features computed from weekly activity patterns. The separation of the clusters demonstrated in the embedding visualization confirms the quality of the clustering results.

### 6.3. City Clusters

**Figure 6:**
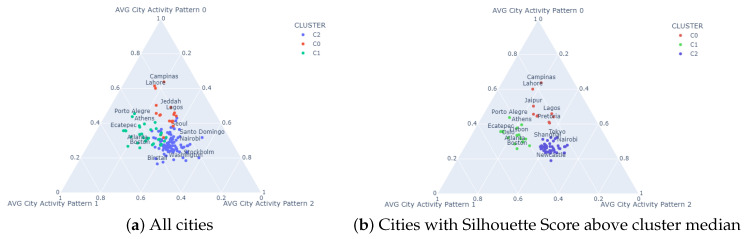
Visualization of city clusters on the convex space determined via the average linear coefficients of each topic representative activity pattern.

Figure 6 visualizes the plot of the cities in the convex region defined by three of the city features, namely the averages of the linear decomposition coefficients of activity patterns, discarding the standard deviations for this visualization. Each color indicates a different cluster. In addition, the names of some cities within each cluster are shown. Thus, it can be seen in Figure 6a that Cluster 0 (C0) includes cities such as Campinas (Brazil), Lahore (Pakistan), Jeddah (Saudi Arabia) and Lagos (Nigeria). Cluster 1 (C1) includes cities such as Porto Alegre (Brazil), Athens (Greece), Ecatepec (Mexico) and Atlanta and Boston (USA). In Cluster 2 (C2), we find cities such as Seoul (South Korea), Santo Domingo (Dominica Republic), Nairobi (Kenya), Stockholm (Sweden), Washington (USA) and Birstall (England). In this figure, separation of the red-colored cluster is clearer than the separation of green and blue clusters.

Enhanced cluster separation is shown in Figure 6b. In this figure, cities were filtered based on their Silhouette Score, leaving only those with a score higher than the median score of each cluster. Thus, we visualize the central core of each cluster, enhancing the separation between them.

**Figure 7:**
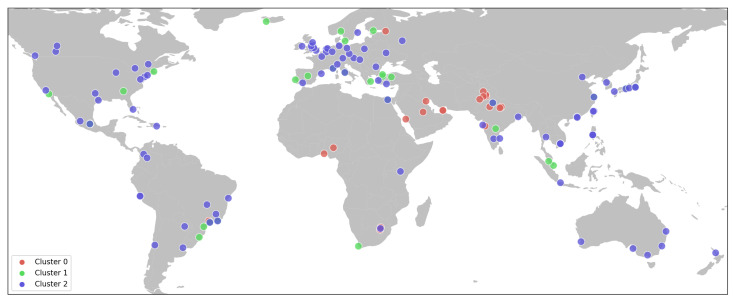
Geographical representation of city clusters.

A geographical representation of the clusters in Figure 7 shows the spatial distribution of the cities that belong to each cluster. The geographical location of Cluster 0 stands out, whose 20 cities are located mainly in the Middle East, South Asia and Africa. The cities corresponding to Cluster 1 and Cluster 2 are distributed in practically the same territories, except that we did not find any of the 32 cities of Cluster 1 in East Asia and Oceania. Finally, Cluster 2 stands out because many of its 92 cities are in central Europe. Cluster 1 appears to include big administrative centers, many of them the country’s capital city.

### 6.4. City Clusters and The City Innovation Index

**Figure 8:**
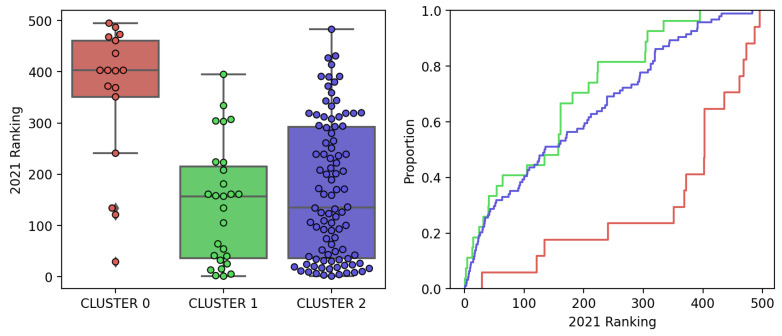
City Innovation Index 2021 distribution over the optimal clusters found via K-Means.

Figure 8 shows the distributions of the City Innovation Index ranking for the year 2021 according to the clusters found in our analysis. Figure 8 (left) is a boxplot where each data point corresponds to a city, indicating the position in the innovation ranking (lower is better). Figure 8 (right) corresponds to the cumulative distribution where population percentage is equated to probability. In this figure, the difference between the cities that form Cluster 0 and the rest of the cities stands out. The cities in Cluster 0 are systematically in the last positions of the innovation ranking and half of the cities in this cluster are in the last quintile of the innovation ranking. Concerning the rest of the clusters, no significant differences between them are observed in the location of their cities in the ranking. In both groups, half of the cities are within the first 150 most innovative cities. Our conclusion from this analysis is that it is possible to discriminate poorly from highly performing cities in the City Innovation Index on the basis of the information provided by the activity patterns of the city.

**Figure 9:**
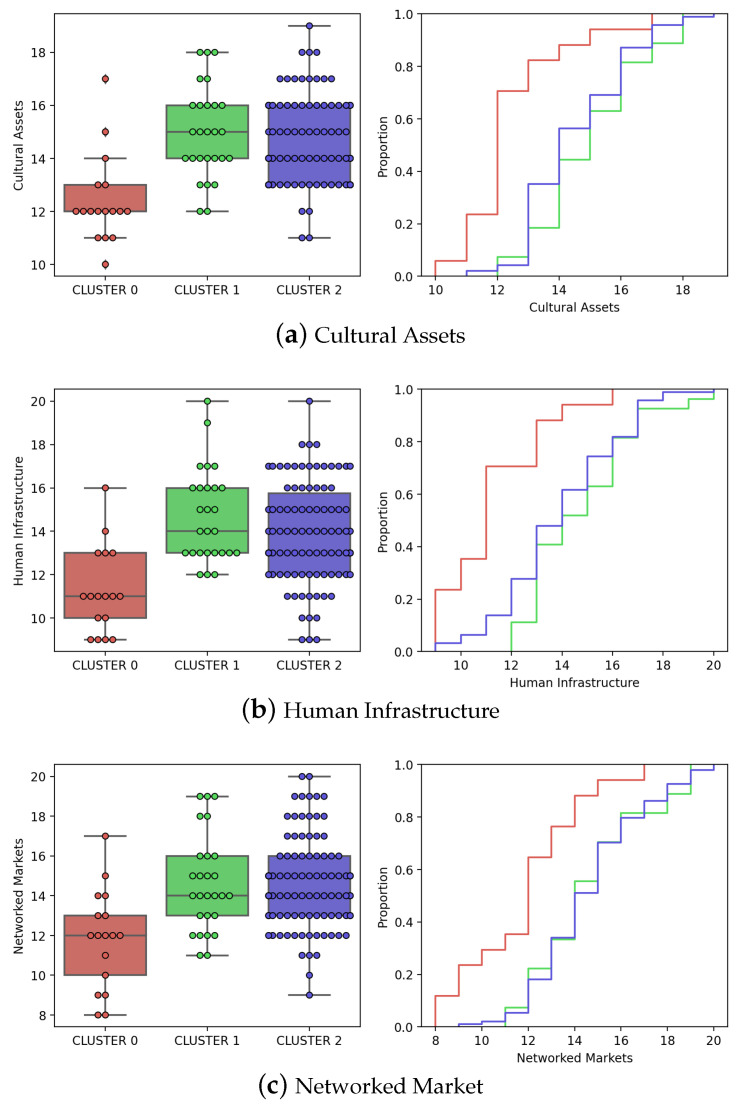
Distribution of innovation disaggregated indices over the optimal clusters found by K-Means.

Additionally, we present the distribution of the factors that make up the City Innovation Index for the cities under study. Figure 9 shows how the Cultural Assets (a), Human Infrastructure (b) and Networked Market (c) indexes are distributed. This disaggregation does not offer variations in interpretation. The cities of Cluster 0 are easily discriminated from the rest in relation to those indices. In this case, these cities have less cultural capital, their infrastructure is also far from world standards and their markets need improvement in order to be sufficiently connected and integrated with the rest of the world. On the other hand, Clusters 1 and 2 show little differences between them when compared based on any of these three indicators.

Finally, Table 3 shows the list of the core cities of each cluster (those with Silhouette Score above the median) ranked by their City Innovation Index. The Table shows the name and country for each city, its rank according to the City Innovation Index and the clustering features given by the average linear decomposition of city weekly activity patterns into topics (Columns T0, T1 and T2).

Regarding Cluster 0, the cities that belong to it have the last ranking positions and the coefficients for topic 0 dominate the other coefficients, i.e., T0 > T1 and T0 > T2. Recalling Figure 2, topic 0 has two different activity patterns of working days and weekends. During the week, the activity of this pattern increases as the day progresses and presents two clearly defined peaks. The first activity peak is observed at noon and then descends to a local minimum at 15:00 h. Afterward, the activity reaches its maximum peak around 21:00 h. The main cities of this cluster are located in Africa (South Africa and Nigeria) and India.

Regarding Cluster 1, the first six cities in the table are in the top 100 for innovation. From the seventh onwards, the cities fall in the center of the innovation ranking. However, some cities from developed countries and sectors such as Japan, the USA and Scandinavia already appear as core cities in this cluster. In this cluster, coefficients for topic 1 dominate the other coefficients, i.e., T1 > T0 and T1 > T2. This topic shows relatively low activity during the week and the most significant activity occurs during the weekend. During the week, the activity increases between 09:00 h and 21:00 h without significant variations in activity during this period. During the weekend, the activity increases from 09:00 h, peaking at 15:00 h. and then declining. This fall is more abrupt on Sunday, leaving little activity until dawn the next day.

Finally, Cluster 2 is made up of the world’s greatest cities. This cluster includes Tokyo, New York, London and Shanghai, among other cities of outstanding global importance. For this same reason, the main cities in this cluster are in the top places in the innovation ranking. In this cluster, coefficients for topic 2 dominate the other coefficients, i.e., T2 > T0 and T2 > T1. This topic also shows different activity patterns during the week working days and on the weekend. During the week, the activity is concentrated between 09:00 h and 18:00 h, with a slight drop in activity around noon. During the weekend, the pattern presents a similar structure. However, after the 09:00 h peak, the activity begins to decline over the rest of the day.

To extend the previous analysis, we delve into the relationship between clusters and city population. Table 4 contains, for each cluster, the joint distribution of population and innovation ranking. Both variables are split into quartiles fitted using the whole dataset. The population is shown for thousands of people, while ranking goes from first to last. It is confirmed that Cluster 0 is mainly composed of cities in the last positions of the ranking, with 76% of them in the last quartile of the ranking. Notice that 35.3% of these low ranking cities have between 3 and 8 million inhabitants. On the other hand, Cluster 1 has 66.6% of its cities positioned in the first two quartiles of the innovation ranking. However, most of these are medium/small cities. Finally, Cluster 2 contains the largest cities in the world, encompassing 83% of the cities in the study. Furthermore, although there are no large concentrations, the mega-cities with the highest level of innovation represent 12.8% of this cluster.

## 7. Discussion

The identification of urban features that can have an impact on the potential for innovation generation as a source for wealth and the general wellbeing of citizens and the financial support of city structures is a matter of concern for city managers, regardless of political affiliation labels [55,56]. The relation of the generation of patents and city wealth measured as the GDP per capita has been established [57] via careful geolocalization of the published patents and fine spatial grid GDP that allows one to compute the metropolitan area GDP with some accuracy. The strong interdependence of scientific production, patent generation and technological productions has already been shown [58,59]; hence, predicting innovation potential from citizen behavior patterns gives additional insights into city potential for sustained growth. The work reported in this paper is in line with works that relate the city innovation value (measured by number of patents) with the activity of people, specifically the mobility of researchers [60] in between companies and the cities that host them. However, we must emphasize that our work does not use information about patents, scientific publications or company localizations in order to produce a prediction of the innovation ranking of the city. As a secondary result, we find that population size induces further discrimination between cities’ innovation rankings in agreement with the literature that recognizes that large cities concentrate innovation resources and results [61,62]. Recent reflections [63] on the sources for sustainable urban innovation point to the salient role of social relation factors, which are indirectly modeled by the activity patterns analyzed in our study that capture the time structure of interactions.

## 8. Conclusions and Further Research

Understanding the interaction between micro-level urban activity patterns and large-scale city characteristics is crucial for policymakers and researchers in designing effective and adequate public policies. The availability of digital data sources, such as social media and mobile phones, has opened up new opportunities for the quantitative analysis of this relationship. This paper reports the clustering of cities on the basis of activity features extracted from topic analyses of micro-level activity data coming from multiple virtual sensors. The study uses a worldwide city dataset containing spatiotemporal activity patterns obtained from geotagged social media data. The Silhouette Score was used to select the optimal clustering results from the comparison of state-of-the-art clustering algorithms. The selected model found three well-defined clusters, achieving a 2.7% greater Silhouette Score than the next-best model. Finally, the paper explores the relation between the identified city clusters and large-scale city characteristics, i.e., the City Innovation Index.

The examination of the distribution of the City Innovation Index for each cluster reveals that it is possible to discriminate low performing cities for middle/top performing cities by their cluster assignment. Therefore, the study establishes a link between micro-level activity patterns and large-scale city characteristics that can be further exploited in order to improve discrimination accuracy and sort the items into finer categories of the city innovation ranking. These findings contribute to the growing literature on using digital traces to support urban planning and public policy, providing insights into the relationship between individual-level urban activities and large-scale city characteristics, such as the city ranking in the City Innovation Index. Future work will refine this study by using different areas within the city as the spatial analysis level instead of the entire city.

## Figures and Tables

**Table 1:** Description of the geotagged digital traces dataset. The Source column provides information about the origin of the geotagged digital traces. The dataset references indicate the sources from which the data were obtained and the Events column shows the total number of records.

Source	Dataset Reference	Events
Brightkite checkins	[45]	1,639,399
Foursquare checkins	[46]	7,515,201
	[47]	1,099,826
Geotagged Images	[48]	4,998,865
Geotagged Tweets	[49]	2,041,262
	[50]	187,802
	[51]	47,337
	[50] (Exact Location)	184,547
	[50] (Inferred Location)	2,604,233
Gowalla checkins	[45]	1,992,082
Weeplaces checkins	[45]	4,176,673
Yelp checkins	[52]	5,695,209

**Table 2:** Comparison of clustering models against the K-Means model with k=3. Clusters indicate the number of clusters extracted. The *p*-value column results from the Wilcoxon test comparing the Silhouette Scores. The Difference column is the percentage increase in the Silhouette Score of K-Means against the comparison algorithm.

Algorithm	*k*	*p*-Value	Difference (%)
Mini Batch K-Means	3	$4.16\times 10^{-4}$	12.0%
Agglomerative Clustering	3	$1.83\times 10^{-3}$	4.3%
Spectral Clustering	3	$3.72\times 10^{-5}$	17.4%
BIRCH	3	$3.88\times 10^{-4}$	15.3%
Gaussian Mixture	3	$1.73\times 10^{-6}$	19.8%
K-Means	2	$9.71\times 10^{-5}$	5.2%
Mini Batch K-Means	2	$3.16\times 10^{-3}$	8.7%
Agglomerative Clustering	2	$5.71\times 10^{-2}$	2.7%
Spectral Clustering	2	$1.40\times 10^{-2}$	3.7%
BIRCH	2	$2.16\times 10^{-4}$	9.0%
Gaussian Mixture	2	$2.41\times 10^{-4}$	12.1%

**Table 3:** List of the core cities within each cluster ordered via the ranking established by the City Innovation Index. Only cities whose Silhouette Score is higher than the cluster median are included. T0, T1 and T2 columns correspond to clustering features as explained in the text.

CLUSTER C0					CLUSTER C1					CLUSTER C2				
City	Ranking	T0	T1	T2	City	Ranking	T0	T1	T2	City	Ranking	T0	T1	T2
Campinas (Brazil)	134	0.63	0.16	0.19	Saitama (Japan)	1	0.31	0.40	0.28	Tokyo (Japan)	1	0.32	0.24	0.43
Vereeniging (South Africa)	372	0.40	0.23	0.35	Boston (USA)	2	0.25	0.47	0.26	New York (USA)	3	0.32	0.27	0.40
Lahore (Pakistan)	403	0.60	0.22	0.17	Atlanta (USA)	13	0.28	0.47	0.24	Sydney (Australia)	4	0.23	0.29	0.46
Pune (India)	436	0.45	0.29	0.24	Oslo (Norway)	25	0.32	0.48	0.19	Dallas (USA)	6	0.28	0.29	0.41
Pretoria (South Africa)	461	0.41	0.24	0.34	Helsinki (Finland)	41	0.33	0.43	0.22	Houston (USA)	8	0.24	0.31	0.44
Lagos (Nigeria)	468	0.45	0.20	0.33	Copenhagen (Denmark)	54	0.29	0.42	0.27	Chicago (USA)	9	0.24	0.31	0.44
Jaipur (India)	473	0.50	0.27	0.22	Guarulhos (Brazil)	134	0.37	0.41	0.20	London (UK)	11	0.24	0.33	0.41
Lucknow (India)	487	0.44	0.28	0.27	Lisbon (Portugal)	158	0.33	0.42	0.23	Shanghai (China)	15	0.30	0.30	0.39
Cawnpore (India)	495	0.44	0.27	0.27	Ecatepec (Mexico)	161	0.35	0.50	0.13	Los Angeles (USA)	20	0.23	0.33	0.42

**Table 4:** City distribution by population and innovation ranking. Each entry within each cluster row shows the distribution by population and innovation ranking quartiles.

		City Innovation Ranking			
Cluster	City Population	(0, 41]	(41, 161]	(161, 311]	(311, 500]
C0	(0–1508]	-	5.9%	5.9%	11.8%
	(1508–3002]	5.9%	-	-	17.6%
	(3002–8154]	-	5.9%	-	35.3%
	(8154–37,977]	-	-	-	11.8%
C1	(0–1508]	11.1%	25.9%	7.4%	3.7%
	(1508–3002]	-	7.4%	7.4%	-
	(3002–8154]	18.5%	-	3.7%	-
	(8154–37,977]	3.7%	-	7.4%	3.7%
C2	(0–1508]	3.2%	6.4%	7.4%	2.1%
	(1508–3002]	2.1%	10.6%	5.3%	9.6%
	(3002–8154]	8.5%	5.3%	5.3%	3.2%
	(8154–37,977]	12.8%	4.3%	7.4%	6.4%

## Data Availability

The data sources are publicly accessible and this article details the references for each dataset. The collated data used in this paper is available from open data repository https://doi.org/10.5281/zenodo.7949307 (accessed on 22 May 2023).
